# Correlation between Targeted qPCR Assays and Untargeted DNA Shotgun Metagenomic Sequencing for Assessing the Fecal Microbiota in Dogs

**DOI:** 10.3390/ani13162597

**Published:** 2023-08-11

**Authors:** Chi-Hsuan Sung, Rachel Pilla, Chih-Chun Chen, Patricia Eri Ishii, Linda Toresson, Karin Allenspach-Jorn, Albert E. Jergens, Stacie Summers, Kelly S. Swanson, Holger Volk, Teresa Schmidt, Helene Stuebing, Johanna Rieder, Kathrin Busch, Melanie Werner, Anja Lisjak, Frederic P. Gaschen, Sara E. Belchik, M. Katherine Tolbert, Jonathan A. Lidbury, Joerg M. Steiner, Jan S. Suchodolski

**Affiliations:** 1 Gastrointestinal Laboratory, School of Veterinary Medicine and Biomedical Sciences, Texas A&M University, College Station, TX 77840, USA; csung@cvm.tamu.edu (C.-H.S.);; 2 Department of Equine and Small Animal Medicine, Faculty of Veterinary Medicine, Helsinki University, 00014 Helsinki, Finland; 3 Evidensia Specialist Animal Hospital, 25466 Helsingborg, Sweden; 4 Department of Veterinary Clinical Sciences, College of Veterinary Medicine, Iowa State University, Ames, IA 50011, USA; 5 Carlson College of Veterinary Medicine, Oregon State University, Corvallis, OR 97331, USA; 6 Department of Animal Sciences, University of Illinois at Urbana-Champaign, Urbana, IL 61820, USA; 7 Department of Small Animal Medicine and Surgery, University of Veterinary Medicine, 30545 Hannover, Germany; 8 Clinic of Small Animal Medicine, Ludwig-Maximilians University, 80539 Munich, Germany; 9 Clinic for Small Animal Internal Medicine, Vetsuisse Faculty, 8057 Zurich, Switzerland; 10 Small Animal Clinic of Veterinary Faculty Ljubljana, University of Ljubljana, 1000 Ljubljana, Slovenia; 11 Department of Veterinary Clinical Sciences, School of Veterinary Medicine, Louisiana State University, Baton Rouge, LA 70803, USA

**Keywords:** canine dysbiosis index, quantitative PCR, shotgun metagenomic sequencing, chronic enteropathy, microbiota

## Abstract

**Simple Summary:**

Untargeted shotgun DNA sequencing of fecal samples is a relatively novel approach to study the microbiome. This method allows better classification of bacteria on a species level compared to traditional 16S rRNA gene sequencing, and provides data about viruses, fungi, archaea, and functional genes. A targeted qPCR-based dysbiosis index has been recently introduced to evaluate the fecal microbiota in dogs. This study evaluated the agreement for core bacterial taxa between targeted qPCR assays and relative abundances obtained by shotgun DNA sequencing. We analyzed the fecal microbiota of 296 dogs with various clinical phenotypes using both methods. Significant correlations were found between the two methods, and the qPCR-based dysbiosis index accurately reflected shifts in the microbiome of dogs as observed by DNA shotgun sequencing.

**Abstract:**

DNA shotgun sequencing is an untargeted approach for identifying changes in relative abundances, while qPCR allows reproducible quantification of specific bacteria. The canine dysbiosis index (DI) assesses the canine fecal microbiota by using a mathematical algorithm based on qPCR results. We evaluated the correlation between qPCR and shotgun sequencing using fecal samples from 296 dogs with different clinical phenotypes. While significant correlations were found between qPCR and sequencing, certain taxa were only detectable by qPCR and not by sequencing. Based on sequencing, less than 2% of bacterial species (17/1190) were consistently present in all healthy dogs (*n* = 76). Dogs with an abnormal DI had lower alpha-diversity compared to dogs with normal DI. Increases in the DI correctly predicted the gradual shifts in microbiota observed by sequencing: minor changes (R = 0.19, DI < 0 with any targeted taxa outside the reference interval, RI), mild-moderate changes (R = 0.24, 0 < DI < 2), and significant dysbiosis (R = 0.54, 0.73, and 0.91 for DI > 2, DI > 5, and DI > 8, respectively), compared to dogs with a normal DI (DI < 0, all targets within the RI), as higher R-values indicated larger dissimilarities. In conclusion, the qPCR-based DI is an effective indicator of overall microbiota shifts observed by shotgun sequencing in dogs.

## 1. Introduction

The gut microbiome plays an important role in health and disease. In dogs, both acute [1,2] and chronic enteropathy [3,4] have been linked to alterations in the gut microbiome. Therefore, understanding the composition and function of the normal gut microbiome is essential for developing new diagnostic tools and therapeutic approaches for diseases and improving our understanding of their underlying mechanisms. Several techniques, such as 16S rRNA gene sequencing, DNA metagenomic shotgun sequencing, metatranscriptomics, fluorescence in situ hybridization, and quantitative PCR (qPCR) can be applied to study the gut microbiome [5,6,7]. These techniques can be categorized into two groups: targeted and untargeted assays.

An untargeted assay, also known as a discovery-based assay, is an approach to detect a broad range of targets without prior knowledge of what might be present in a sample. These assays are useful to generate comprehensive profiles of complex biological samples, for example the fecal microbiota. The most commonly used untargeted methods are 16S rRNA gene sequencing [8,9,10] and DNA shotgun metagenomic sequencing. The former technique provides an overview of bacterial communities up to the genus level, whereas the latter provides bacterial taxonomy up to the species and strain-level resolution [11,12,13]. Amplifying specific gene regions is the first step in 16S rRNA sequencing. However, the choice of primer sets can lead to variable results with different levels of amplification bias. In contrast, shotgun metagenomic sequencing does not involve gene amplification but instead breaks down DNA into fragments for sequencing, allowing for a more accurate estimation of abundance. Both sequencing methods lack analytical validation and reference intervals are unavailable, hindering comparison of results between runs [14]. Furthermore, even though standardized protocols have been proposed [15], batch effects are inevitable when using untargeted assays. Additionally, analyzing sequencing data is computationally demanding and requires specialized skills and expertise. Nevertheless, sequencing is a powerful discovery tool for characterizing microbial communities.

Targeted assays measure a predefined set of objectives, in this case, specific microbes. A quantitative PCR (qPCR)-based assay, called the dysbiosis index (DI), has been developed to evaluate the fecal microbiota in dogs [16]. The DI is designed to quantify a particular group of clinically relevant core bacterial taxa and total bacterial abundance, which are commonly altered in dogs with chronic enteropathy (CE) and has shown utility as a functional marker of intestinal health in a recent meta-analysis [17]. During qPCR, a DNA template is amplified in the presence of specific primers to quantify a known target in a sample. Such qPCR assays can be highly reproducible, sensitive, and specific, and are time and cost-effective. However, they require prior knowledge of the microbial targets of interest and cannot detect unexpected or novel microbial taxa.

Both untargeted and targeted assays have their strengths and limitations. Combining the strengths of both approaches enhances our understanding of the gut microbiome and its role in health and disease. The aim of this study was to evaluate the correlation between untargeted DNA shotgun sequencing, targeted qPCR assays, and the qPCR-based canine DI in dogs with a wide range of different clinical phenotypes, as well as to evaluate whether the qPCR-based DI can accurately reflect global shifts in the gut microbiome.

## 2. Materials and Methods

### 2.1. Study Population

This study included fecal samples from 296 dogs with diverse clinical phenotypes, sourced from previous studies. The study population consisted of 78 clinically healthy control dogs, 146 dogs with chronic enteropathy (CE), 35 dogs with diseases unrelated to the gastrointestinal tract (22 dogs with neurological signs and 13 dogs with non-GI neoplasia), 20 dogs on antibiotics, and 17 dogs with acute diarrhea (AD).

Clinically healthy control dogs did not receive any antibiotics, antacids, anti-inflammatory medications, or corticosteroids within the past 6 months. The clinical workup of dogs with acute or chronic GI signs followed standardized protocols described in Werner et al. [18] and Toresson et al. [19]. Briefly, the inclusion criteria for dogs with CE were dogs that presented with GI signs (i.e., vomiting, diarrhea, hyporexia/anorexia, and/or weight loss) for at least three weeks. Dogs with CE that had any recorded antibiotic exposure were excluded from the study. The inclusion criteria for dogs with AD were dogs presenting acute GI signs, such as vomiting or diarrhea, for fewer than three days, and fecal samples were collected upon presentation before any treatments.

Among the 20 dogs receiving antibiotics, sixteen were healthy dogs and had received metronidazole during an experimental trial with antibiotic exposure [20,21]. The other four healthy dogs had exposure to antibiotics documented in the medical history (as reported by the owners), but the reason for treatment and the exact antimicrobial type were unknown.

The fecal samples used in this study were obtained from various previous studies collected at different institutions and hospitals (Supplementary Table S1). Upon collection, all samples were stored at either −20 °C or −80 °C and later transported in bulk with dry ice to a central laboratory (Gastrointestinal Laboratory at Texas A&M University) for processing and subsequent storage at −80 °C.

### 2.2. Quantitative PCR and Dysbiosis Index (DI)

DNA was extracted from an aliquot of 100–120 mg fecal sample using a bead-beating method with a MoBio Power soil DNA isolation kit. The qPCR assays were applied to quantify total bacteria, *Blautia, Clostridium* (*Peptacetobacter*) *hiranonis, Escherichia coli, Faecalibacterium, Fusobacterium, Streptococcus*, and *Turicibacter*. The qPCR assays have been described previously [16] and in Supplementary Table S1. Briefly, the qPCR assays were performed in the following order: at 95 °C maintained for 2 min, 40 cycles at 95 °C for 5 s, and then annealing at the optimized temperature for 10 s, using 10 μL of SYBR-based reaction mixtures (5 μL of SsoFast™ EvaGreen^®^ supermix [Bio-Rad Laboratories GmbH, Düsseldorf, Germany]), 1.6 μL of high-quality PCR water, 0.4 μL of each primer (final concentration: 400 nM), and 2 μL of DNA. Both positive and negative controls were included for all qPCR assays to ensure the accuracy and reliability of the results.

The DI was calculated based on the results of the qPCR assays using a previously described algorithm [16]. Furthermore, we further divided the samples into four groups based on the currently used clinical classification of the DI. A DI < 0 and with all targeted taxa within the reference interval (RI) was considered normal. A DI < 0 but with any of the targeted taxa outside the RI was defined as minor shift in the microbiome. A DI between zero and two was defined as mild to moderate microbiome shift. A DI > 2 was classified as significant dysbiosis.

In addition to the bacterial groups targeted in the DI, additional bacterial taxa that were found highly abundant (a maximum relative abundance >50% and/or median relative abundance >1%) in healthy control dogs upon metagenomic sequencing were also quantified by qPCR assays in a subset of healthy dogs (selected based on DNA availability) to allow correlation between both methods. The genera *Bacteroides* and *Bifidobacterium* were quantified by qPCR assays in 78 of healthy control dogs. Genus *Collinsella, Prevotella copri,* and *Ruminococcus gnavus* were quantified by qPCR assays in 37/78 of the healthy control dogs. Primers and other qPCR information for the additional targets are summarized in supplementary Table S2 and in previous studies [22,23]. The qPCR assays were similar to those mentioned above with optimal annealing temperature and time: 60.3 °C for 5 s for genus *Collinsella*, 58.4 °C for 5 s for *P. corpi*, and 60.0 °C for 15 s for *R. gnavus*.

### 2.3. Shotgun Metagenomic Sequencing

The DNA shotgun metagenomic sequencing was performed at Diversigen (New Brighton, MN, USA). Libraries were prepared with a procedure adapted from the Nextera XT kit (Illumina, San Diego, CA, USA). Libraries were sequenced on an Illumina NovaSeq 6000 using paired end 2 × 150 reads with a mean target depth of 2M reads/sample (Illumina). Both positive and negative controls were included on each DNA extraction plate as well as on each library preparation plate. DNA sequences were filtered for low quality (Q-Score < 30) and length (<50), and adapter sequences were trimmed using Cutadapt. Host sequences were removed using Bowtie2. Sequences were trimmed to a maximum length of 100 bp before alignment and converted to a single fasta using shi7. DNA sequences were aligned to a curated database containing all representative genomes in RefSeq for bacteria with additional manually curated strains (DivDB-Canine). Alignments were made at 97% identity against all reference genomes. Every input sequence was compared to every reference sequence in Diversigen’s DivDB-Canine database using fully gapped alignment with BURST. Ties were broken by minimizing the number of unique Operational Taxonomic Units (OTUs). Each input sequence was assigned the lowest common ancestor consistent across at least 80% of all reference sequences tied for the best hit for taxonomy assignment. OTUs accounting for less than one-millionth of all species-level markers and those with less than 0.01% of their unique genome regions covered (and <1% of the whole genome) were discarded. The number of counts for each OTU was normalized to the average genome length. Count data were then converted to relative abundance for each sample. The normalized and filtered tables were used for all downstream analyses.

For downstream analysis, QIIME 22021.11 was applied. The data was analyzed on two different rarefaction levels. To account for the variable sequencing count per sample, samples were rarefied with the lowest reads of 9788 so that all samples could be included. To increase the detection rate of taxa, rarefaction depth of 100,000 was also applied, which resulted in the exclusion of a subset of samples (*n* = 11). Alpha diversity metrics Shannon, Chao1, and observed features were calculated on both rarefaction levels. Beta-diversity was evaluated by the Bray–Curtis distance by visualization with principal coordinate analysis plots.

Metagenomic sequences are available under BioProject ID PRJNA975215.

### 2.4. Statistical Analyses

The Spearman test was used to evaluate the correlations between the abundance of taxa obtained by qPCR and the relative abundance acquired by sequencing. The Bonferroni method was applied to adjust the *p*-values for multiple comparisons. Alpha diversity metrics were compared between different groups based on different DI classifications using Kruskal–Wallis tests, followed by Dunn’s tests. Beta diversity between groups was analyzed with the analysis of similarity tests, ANOSIM, using Primer 7 (Plymouth Routines in Multivariate Ecological Research Statistical Software, v7.0.13). For correlating these global microbial shifts between DI and sequencing using ANOSIM, only the original DI containing the original taxa was used. Statistical significance was set at *p* < 0.05.

## 3. Results

### 3.1. Dysbiosis Index of the Study Population

**Figure 1:**
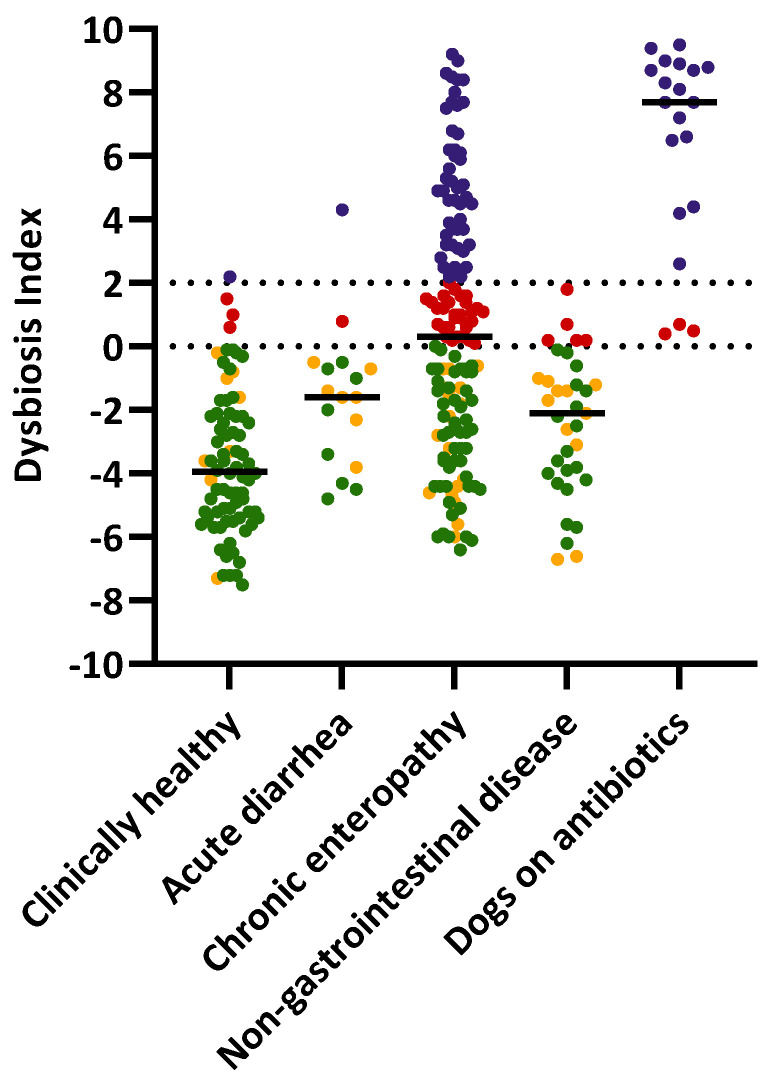
Scatter plot of dysbiosis index in clinically healthy dogs, dogs with chronic enteropathy, dogs with acute diarrhea, dogs with non-gastrointestinal disease, and dogs on antibiotics. Samples are colored based on the subclassification of the DI. Green: normal, DI < 0 with all taxa within reference interval; Yellow: minor changes, DI < 0 with any bacterial taxa out of reference interval; Red: 0 < DI < 2; Purple: DI > 2.

Figure 1 shows the distribution of the DI among dogs with different phenotypes. Table 1 shows the study population (*n* = 296) categorized into four interpretations of the DI.

### 3.2. Alpha Diversity of the Fecal Microbiota in the Study Population

The median sequencing count obtained was 1,248,309 (range: 9788–5,662,490), with one sample having a count as low as 9788, ten samples with counts between 10,000 and 100,000, 96 samples with counts between 100,000 and 1,000,000, and 189 samples with counts higher than 1,000,000.

**Figure 2:**
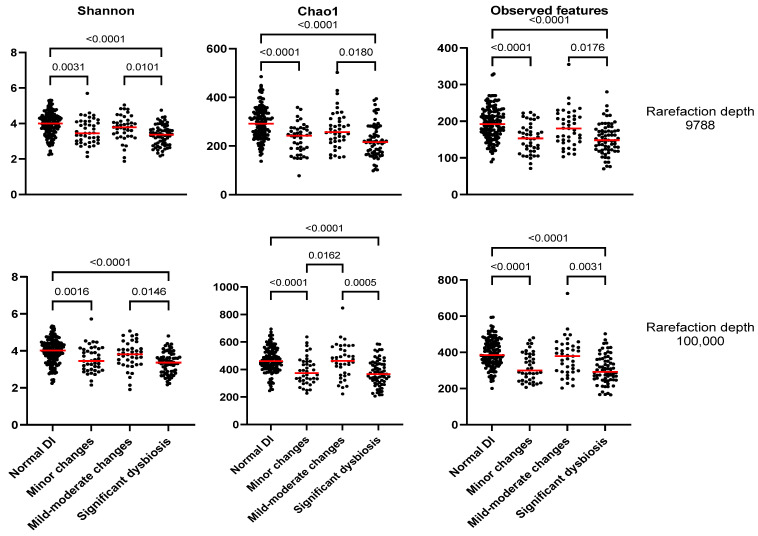
Alpha diversity metrics in dogs with normal DI (DI < 0), minor changes (DI < 0 with any taxa out of reference interval), mild to moderate changes (0 < DI < 2), and significant dysbiosis (DI > 2). The rarefaction depth was set at 9788 (on the top) or 100,000 (at the bottom).

Figure 2 displays the alpha diversity metrics. Dogs with a normal DI had significantly higher (*p* < 0.0001) alpha-diversity metrics than dogs with minor changes and significant dysbiosis. With an increase in the rarefaction depth from 9788 to 100,000, the Shannon index (richness and evenness) remained constant, while Chao1 and observed features (richness) increased 1.5- to 2-fold. However, the pattern between the four groups was similar for all indices, regardless of the difference of the rarefaction depth. Chao1 in dogs with minor changes was significantly higher than dogs with mild to moderate changes only at the higher rarefaction depth. However, a considerable degree of overlap in values between the different groups was observed.

**Figure 3:**
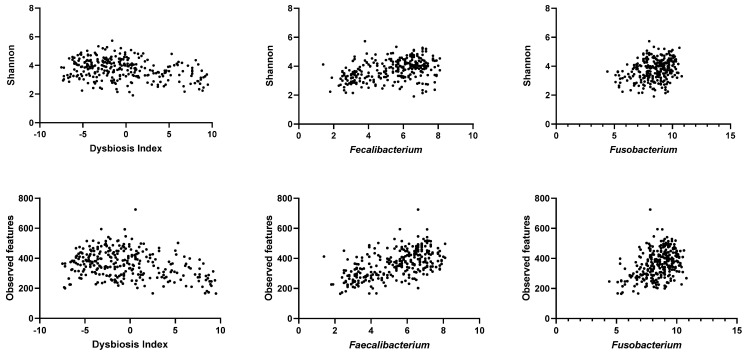
Representative figures of the correlation between alpha diversity metrics (Shannon on the top and observed features at the bottom) by metagenomic shotgun sequencing and dysbiosis index (**left**), abundance of *Faecalibacterium* by qPCR (**middle**), and abundance of *Fusobacterium* by qPCR (**right**). *p*-values and R-values are listed in Table 2.

Table 2 and Figure 3 show the correlations between the DI and the alpha diversity metrics at the rarefaction depth of 100,000. The DI was negatively correlated with Shannon, Chao1, and observed features. Conversely, abundances of *Faecalibacterium*, *Fusobacterium*, and *C. hiranonis* were positively correlated with all alpha diversity metrics. However, the abundances of *Blautia* and *Turicibacter* were only significantly correlated with observed features and Chao1, but not Shannon. Notably, the abundances of *Streptococcus* and *E. coli* were not correlated with any of the alpha diversity metrics.

### 3.3. Beta Diversity of the Fecal Microbiota in the Study Population

**Figure 4:**
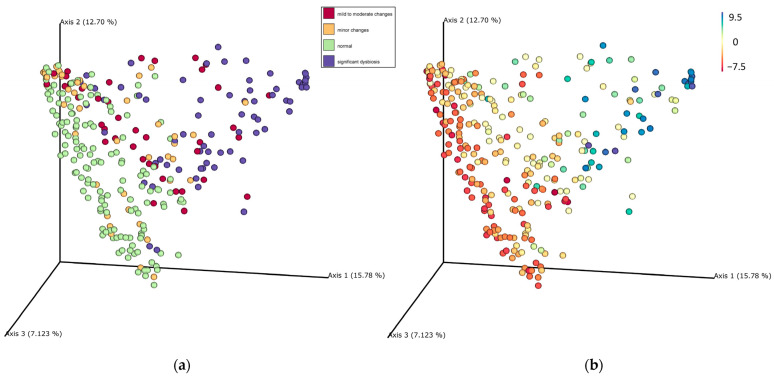
Principal Component Analysis (PCA) plot based on Bray–Curtis distance derived from the sequencing data. (**a**) Samples are color-coded based on the interpretation of the dysbiosis index. Purple: Significant dysbiosis (DI > 2); Red: Mild to moderate change (0 < DI < 2); Yellow: Minor changes (DI < 0 with any bacterial taxa out of the reference interval); Green: Normal (DI < 0 with every taxon within the reference interval). Statistical analysis is shown in Table 3. (**b**) Samples are color-coded based on the value of the DI, with the gradient of red to blue representing the value of DI from low to high. Statistical analysis is shown in Table 4.

Figure 4 presents the beta diversity based on Bray–Curtis. Dogs with minor changes and increased DI (>0) clustered away from dogs with a normal DI. According to ANOSIM tests, the R values (where higher R-values indicate larger size effects) increased proportionally with an increase in the DI, with the highest R value found in dogs with DI > 8 (Table 3). Among dogs with DI < 0, no differences (*p* = 0.56) were found between dogs with −5 < DI < −10 and dogs with −5 < DI < 0 (Table 4). Supplementary Figure S1 shows the plot of beta diversity between different disease phenotypes.

### 3.4. Correlation between qPCR-Based Dysbiosis Index and Shotgun Metagenomic Sequencing Data

**Figure 5:**
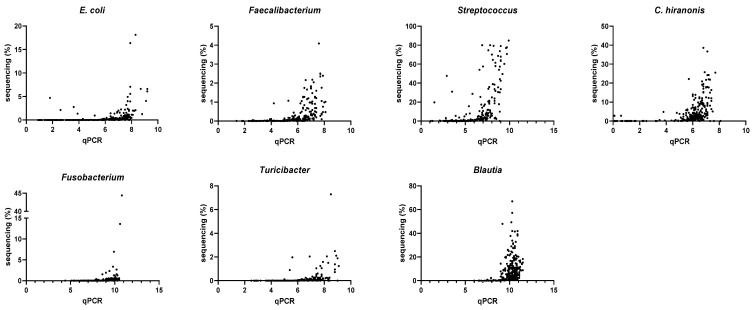
Correlation between log DNA by qPCR-based dysbiosis index (DI) and the relative abundance by shotgun metagenomic sequencing. The rarefaction depth was set at 100,000 for the relative abundance.

The abundances of all bacterial groups targeted in the DI were significantly correlated (*p* < 0.001) with the relative abundances acquired by shotgun sequencing (Table 5 and Figure 5). With an increase in the rarefaction depth from 9788 to 100,000, the Spearman’s R-values increased in all groups, but with minimal changes. However, it should be noted that many bacterial groups were undetectable by shotgun metagenomic sequencing in a subset of samples (Table 5).

### 3.5. Core Microbiota in Healthy Dogs

Descriptive data of core bacterial groups in healthy control dogs are shown in Table 6. The median percentage of the total abundance of bacterial groups that are targeted by the DI account for 11.2% (range: 0.5–74.0%). In healthy dogs, the maximum relative abundance of *Lactobacillus*, *Collinsella, Prevotella, Bifidobacterium*, and *Streptococcus* were above 50%, while the minimum relative abundance could be as low as zero, meaning undetectable. For example, *E. coli* was undetectable in 68% and 53% of healthy dogs when the rarefaction depth was set at 9788 (*n* = 78) or 100,000 (*n* = 76), respectively. On the contrary, shotgun metagenomic sequencing was able to identify *C. hiranonis* in all healthy dogs. However, the relative abundance of *C. hiranonis* ranged from 0.01 to 37% in the sequencing data, whereas the absolute quantification by qPCR showed a relatively narrow range with 97% of the healthy dogs within RI (log DNA: 5.1–7.1).

At the rarefaction depth of 100,000, less than 5% of genera (15/328) were found in all healthy dogs (*n* = 76). These genera were *Bacteroides, Blautia, Clostridium, Coprococcus, Eubacterium, Fusicatenibacter, Lachnoclostridium*, *Roseburia*, each of one unknown genus in the families *Erysipelotrichaceae*, *Lachnospiraceae*, and *Peptostreptococcaceae*, orders Bacteroidales and Clostridiales, and two unclassified genera. Similarly, less than 2% of species (17/1190) were detectable in all healthy dogs. These species are presented in Table 7. At the species level, a substantial proportion of bacterial groups (12.8%, 152/1190) were unclassified.

At the rarefaction depth of 1,000,000, this analysis included 46 healthy dogs, with 31 of them being excluded due to having counts below 1 million. Around 10% of genera (28/272) and 5% of species (40/852) were detected among this group of 46 healthy dogs.

## 4. Discussion

Our study demonstrated a robust correlation between qPCR targeting core bacteria taxa, the dysbiosis index (DI), and metagenomic sequencing data. Higher DI values indicated a more pronounced deviation from the healthy reference group, which was supported by ANOSIM analysis on beta diversity showing an increasing R value as the DI value increased. Additionally, an increase in DI was correlated with a decrease in alpha-diversity. These findings confirm that the DI accurately reflects the extent of shift in the overall fecal microbiota composition in dogs.

The DI was negatively correlated with Shannon, Chao1, and observed features, indicating that dogs with a higher DI tend to have decreased alpha diversity, characterized by low richness and evenness of the bacterial communities. In contrast, the abundances of *Faecalibacterium*, *Fusobacterium*, and *C. hiranonis* were positively correlated with alpha diversity metrics, suggesting that higher abundance of these bacterial groups reflects higher microbial diversity. These bacterial groups had been reported to be beneficial in dogs, as *Faecalibacterium* [24] and *Fusobacterium* [25] produce short-chain fatty acids, which have anti-inflammatory and immunomodulatory properties [26]. *C. hiranonis* is linked to conversion of primary to secondary bile acids in dogs, which is important in the regulation of *C. difficile* and *C. perfringens* in both dogs and humans [27,28,29]. In humans, microbially derived secondary bile acids have been reported to inhibit the growth and germination of *C. difficile* [30]. Similarly, secondary bile acids inhibited growth of *E. coli* and *C. perfringens* isolates from dogs in vitro [31]. The loss of *C. hiranonis* and the expansion of primary fecal bile acids has been repeatedly reported in dogs with CE [32,33] and dogs receiving antibiotics [20,34]. Bile acid dysmetabolism and dysbiosis were also found in humans with inflammatory bowel disease [35]. Additionally, previous studies using 16S rRNA sequencing have reported the correlations between alpha diversity and both DI and *C. hiranonis* [36]. However, the abundances of *Blautia* and *Turicibacter* were only significantly correlated with observed features and Choa1, but not Shannon, indicating that these bacterial groups may contribute more to the richness rather than the evenness of the bacterial community. It is important to note that the correlations between alpha diversity metrics and the targeted bacterial groups in the DI were statistically significant but weak. However, as shown in Supplementary Figure S2, the differences in alpha diversity between phenotypes were also overlapping and no significant different were found between healthy dogs and dogs with any of the disease phenotypes.

The PCA plot based on the Bray–Curtis distances showed that dogs with significant dysbiosis had a microbiota shift far away from dogs with a normal DI, indicating a marked difference in the overall microbiota composition. Moreover, the increasing R values shown in the ANOSIM analysis also indicated that the dogs with a higher DI had larger shifts compared to dogs with lower DIs. This finding was in line with studies using 16S rRNA gene sequencing, where dogs with normal DI clustered away from dogs with an increased DI on the PCA plot on Bray–Curtis distances [36] or UniFrac distances [20].

The significant correlation between a targeted qPCR-based DI and an untargeted metagenomic shotgun sequencing found in this study could be attributed to the process used to develop the DI. During the development of the DI, an initial set of bacterial phyla (*Proteobacteria, Firmicutes, Fusobacteria, Bacteroidetes,* and *Ruminococcaceae*) and genera (*Bifidobacterium, Blautia*, *Faecalibacterium*, *Turicibacter*, *Lactobacillus*, *C. perfringens*, *C. hiranonis*, and *E. coli*) were selected based on results from studies using 16S rRNA gene sequencing and/or qPCR, and quantified by qPCR for feature selection to identify the best combination that would differentiate the fecal microbiota in dogs with CE from healthy dogs [16]. The DI model ultimately consisted of seven bacterial groups that provided the balance between highest classification accuracy and the lowest number of assays. The DI’s ability to capture the overall shifts in the fecal microbiota is further supported based on the results of the current study.

Consistent abnormal DI values may indicate an imbalance in the gut microbiota and reflect more severe abnormalities within the gastrointestinal tract. Dogs with a DI above zero were found to cluster farther away from dogs with a DI below zero on the PCA plot based on Bray–Curtis distances, indicating a significant shift in the fecal microbiota as assessed by metagenomics. A higher DI might indicate larger shifts in the microbiota, which was also evident in a recent study that demonstrated that dogs with CE and a higher DI had a worse response to fecal microbiota transplantation as an adjunct treatment [19]. The DI has been used as a monitoring tool to assess whether the microbiota returns to a normal state or improves in response to treatment. For instance, healthy dogs receiving antibiotics had a significantly increased DI which decreased over time after the antibiotic was discontinued [20,34].

Notably, not all dogs with CE in our study population exhibited shifts in sequencing and DI. Approximately 36% of dogs with CE had a normal DI in this study, consistent with findings from other studies [19,32,37,38,39]. This result was confirmed by sequencing, as these dogs with CE and a normal DI clustered together with the healthy dogs with a normal DI in the PCA plot based on Bray–Curtis distances (Supplementary Figure S1). It is possible that these dogs with CE and a normal DI may have a different pathophysiology compared to dogs with CE and dysbiosis. Canine CE is known to be a multifactorial disease, where the microbiota is only one of the associated factors. Future studies on dogs with CE and a normal DI are necessary, as these dogs may have different therapeutic needs and/or prognostic compared to dogs with CE and dysbiosis.

Sequencing approaches provide an overview and serve as an initial step in gathering information on microbiota composition in a specific disease or condition. However, the untargeted nature of this technique can make reproducibility difficult to define. Comparing sequencing results over time or between studies that use different sequencing platforms requires caution. The calculation of a relative abundance could be influenced by factors such as sequencing depth, data normalization, and data processing methods [40]. As a result, different studies might report different relative abundances for the same taxa in a group with similar conditions. Furthermore, using relative abundances often leads to misinterpretation, as the increase of one taxon leads to the concurrent decrease of the other(s) within compositional data [14,41].

Using qPCR assays allowed for a quantitative approach and the establishment of reference intervals for the DI and each target taxon. In contrast, the metagenomic sequencing results revealed a wide range of major and prevalent bacterial genera, such as *Collinsella, Bacteroides, Prevotella, Streptococcus, Lactobacillus*, and *Bifidobacterium*, in healthy dogs. While these genera are expected to be present in all healthy dogs, this study detected zero counts for many bacterial genera using shotgun sequencing. However, as qPCR uses specific primer sets to identify these bacterial groups, most of these prevalent bacterial groups were detectable by qPCR in all healthy dogs. Specifically, *Collinsella, Bacteroides, Prevotella*, and *Bifidobacterium* were detected by qPCR in 100% and *Streptococcus* was detected in 97% of healthy dogs. These findings were not consistent within the sequencing approach. For instance, the relative abundance of the genus *Collinsella* ranged from 0 to 83%. It is unlikely that *Collinsella* accounts for 83% of the fecal microbiota in one healthy dog but 0% in another dog. Moreover, it is unlikely that a certain genus accounts for more than 80% of bacterial composition in any healthy dogs. To confirm our untargeted sequencing findings, a qPCR assay was applied and found a narrow range (13.2–15.0 log DNA) of the fecal abundance of *Collinsella* in healthy dogs. This pattern of a wide range of relative abundance in metagenomic sequencing and a narrow range of the qPCR results were also observed in other genera, such as *C. hiranonis* and *R. gnavus*.

In this study, shotgun sequencing yielded varying counts in each sample, ranging from around ten thousand to 5.6 million. This variability may have been caused by technical issues (i.e., bioinformatic pipelines, library preparation, choice of database, etc.), poor quality of DNA, or biological variation in the samples, resulting in fewer or lower- quality reads. Having different count numbers does not necessarily indicate a problem or bias, but accounting for the sequencing depth between samples when interpreting the data is crucial [40]. Rarefaction is often applied in sequencing analysis to address these differences in sequencing counts between samples, but not without controversy [42,43]. Rarefaction randomly subsamples the sequencing counts to an equal number across all samples, to compare the diversity and richness of different samples, when accounting for differences in sequencing depth. For example, Sample A has 2 million counts and Sample B has 0.1 million counts. If the rarefaction depth is set at 0.1 million counts, the process will randomly subsample 0.1 million counts from Sample A and include all counts from Sample B. However, rarefaction has the potential to introduce bias [43]. Altering the rarefaction depth had different impacts on the alpha diversity metrics in this study. Increasing the rarefaction depth by a factor of 10 resulted in minimal changes to the Shannon index, which is an indicator of species diversity and evenness in a sample. However, both the observed features and Chao1 metrics, which estimate the number of unique species present in a sample, increased almost two-fold. Therefore, direct comparisons on alpha-diversity between studies are not appropriate, especially if rarefaction depth is not the same.

Increasing the sequencing rarefaction deptSh reduced the rate of undetectable genera, but also excluded more samples with counts below the rarefaction depth. In this study, 11 samples were excluded for a larger rarefaction count. If a rarefaction depth of 1 million reads per sample had been applied, 108 samples (36%) would need to be excluded from the study, which could be problematic for studies with small sample sizes, or when paired analysis would require the exclusion of study subjects when one sample is lost. Increasing the sequencing depth is a viable solution to obtaining higher read counts in samples, but it comes at a higher financial cost [44]. Moreover, increasing sequencing depth can also lead to a higher rate of sequencing errors, which can affect downstream analysis.

The main advantage of a targeted assay, such as a qPCR-based DI used in this study, is the ability to detect and quantify a pre-defined set of microbial targets with high reproducibility. We were also able to demonstrate that the DI reflects the extent of shifts in the gut microbiome, as observed by shotgun metagenomic sequencing. While the qPCR-based DI provides information about fewer taxa compared to sequencing, it targets the core bacteria and offers advantages such as cost-effectiveness, easy repeatability, and faster turnaround time (can be performed in one day). It also allows comparison of data across studies. Untargeted sequencing techniques, while providing an overview of the microbial community and identifying novel and unexpected microbial taxa, can be more expensive, computationally intensive, and require larger sample sizes to achieve statistical significance compared to targeted assays. This can limit their use in research studies. Nonetheless, untargeted metagenomic sequencing is essential for identifying microbial taxa that may be relevant to disease and provide a comprehensive understanding of the microbiome, such as *R. gnavus* and *P. copri* which were found to be highly abundant in healthy dogs in this study. As for the bacterial taxa not detected by shotgun metagenomics, this could be due to various factors, including differences in assay sensitivity and sequencing depth. Indeed, an increased sequencing depth might have provided more insights, but the current depth was sufficient to establish significant correlations. It is important to consider the advantages and limitations of both targeted and untargeted approaches when selecting the appropriate method for a specific research question, and ideally both methods should be applied to allow for stronger conclusions in research studies.

## 5. Conclusions

This study demonstrated robust correlations between untargeted metagenomic sequencing and targeted qPCR assays. The qPCR-based canine dysbiosis index accurately predicted shifts in the microbiome observed on shotgun metagenomic sequencing. It is important to note that targeted assays, such as qPCR, have limitations as they only detect a pre-defined set of microbial targets. Nonetheless, this study provided evidence for the use of the DI as an effective indicator of shifts in the fecal microbiota in dogs, which allows better comparisons across studies and individual dogs over time due to superior reproducibility and analytical sensitivity. Combining the strengths of both approaches can enhance our understanding of the gut microbiome and its role in health and disease.

## Figures and Tables

**Table 1:** Number and percentage of dogs categorized based on the interpretation of the dysbiosis index (DI) within each clinical phenotype.

Group	Normal	Minor Changes	Mild to Moderate Changes	Significant Dysbiosis	Total
Clinically healthy	66 (85%)	8 (10%)	3 (4%)	1 (1%)	78
Chronic enteropathy	52 (36%)	17 (12%)	29 (20%)	48 (33%)	146
Acute diarrhea	8 (47%)	7 (41%)	1 (6%)	1 (6%)	17
Non-gastrointestinal disease	19 (54%)	11 (31%)	5 (14%)	0 (0%)	35
On antibiotics	0 (0%)	0 (0%)	3 (15%)	17 (85%)	20
Total	145	43	41	67	296

**Table 2:** Correlation between log DNA by qPCR-based dysbiosis index (DI) and the selected alpha-diversity metrics (Shannon and observed features) at the rarefaction depth of 100,000 by shotgun metagenomic sequencing (*n* = 285). Spearman R value and its 95% confidence interval is described. Bolded *p* values indicate statistical significance.

Bacterial Groups	Shannon	*p* Value	Adjusted *p* Value	Observed Features	*p* Value	Adjusted *p* Value
DI	−0.23 (−0.34 to −0.11)	**<0.0001**	**0.0008**	−0.26 (−0.37 to −0.14)	**<0.0001**	**0.0008**
*Faecalibacterium*	0.38 (0.27–0.47)	**<0.0001**	**0.0008**	0.56 (0.47–0.64)	**<0.0001**	**0.0008**
*Fusobacterium*	0.28 (0.16–0.38)	**<0.0001**	**0.0008**	0.36 (0.25–0.46)	**<0.0001**	**0.0008**
*Clostridium hiranonis*	0.16 (0.05–0.28)	**0.005**	**0.04**	0.26 (0.14–0.37)	**<0.0001**	**0.0008**
*Turicibacter*	0.11 (−0.007 to 0.23)	0.06	0.48	0.28 (0.16–0.39)	**<0.0001**	**0.0008**
*Blautia*	0.10 (−0.02 to 0.22)	0.08	0.64	0.25 (0.14–0.36)	**<0.0001**	**0.0008**
*Streptococcus*	−0.08 (−0.19 to 0.04)	0.19	1.0	−0.08 (−0.20 to 0.04)	0.15	1.0
*Escherichia coli*	−0.11 (−0.22 to 0.01)	0.07	0.56	0.02 (−0.10–0.13)	0.79	1.0

**Table 3:** ANOSIM (Analysis of Similarity) test results for the dissimilarity of Bray–Curtis distance between each group and dogs with normal DI. A larger R value indicates a larger difference between the groups.

Compared to Normal Groups	R Value	*p* Value
minor changes	0.19	0.001
mild to moderate changes (0 < DI <2)	0.24	0.001
significant dysbiosis (2 < DI < 5)	0.54	0.001
significant dysbiosis (5 < DI < 8)	0.73	0.001
significant dysbiosis (DI > 8)	0.91	0.001

**Table 4:** ANOSIM test results for the dissimilarity of Bray-Curtis distance between each group and dogs with −5 < DI < −10. A larger R value indicates a larger difference between the groups.

Compared to −5 < DI < −10	R Value	*p* Value
−5 < DI < 0	−0.01	0.56
0 < DI < 2	0.12	0.001
2 < DI < 5	0.30	0.001
5 < DI < 8	0.65	0.001
DI > 8	0.89	0.001

**Table 5:** Correlation between log DNA by qPCR-based dysbiosis index (DI) and the relative abundance by shotgun metagenomic sequencing. Spearman R value and its 95% confidence interval is described. All *p*-values are <0.0001, where adjusted *p*-values are 0.007.

Bacterial Groups Targeted in DI	Spearman R (*n* = 296) Rarefaction Depth of 9788	Spearman R (*n* = 285) Rarefaction Depth of 100,000
*Escherichia coli*	0.80 (0.76–0.84)	0.84 (0.80–0.87)
*Faecalibacterium*	0.80 (0.75–0.84)	0.82 (0.78–0.86)
*Streptococcus*	0.77 (0.71–0.81)	0.77 (0.72–0.82)
*Clostridium hiranonis*	0.73 (0.67–0.78)	0.74 (0.68–0.79)
*Fusobacterium*	0.71 (0.65–0.76)	0.78 (0.72–0.82)
*Turicibacter*	0.65 (0.57–0.71)	0.72 (0.65–0.77)
*Blautia*	0.46 (0.36–0.55)	0.49 (0.39–0.57)

**Table 6:** Descriptive data (median and range) of the abundances of major bacterial groups in healthy dogs by shotgun metagenomic sequencing (relative abundance%) or qPCR (log DNA).

Bacterial Taxa	Sequencing Rarefaction 9788 (*n* = 78)			Sequencing Rarefaction 100,000 (*n* = 76)			qPCR (*n* = 78)		
	Median	Range	UDL ^1^ (%)	Median	Range	UDL ^1^ (%)	Median	Range	UDL ^1^ (%)
*Collinsella*	6.1	0–83.2	1.3	5.9	0–83.6	1.3	13.7	13.2–15.0 ^2^	0
*Blautia*	5.9	0.4–42.1	0	6.3	0.3–41.9	0	10.4	9.1–11.1	0
*Bacteroides*	5.4	0–63.4	33.3	4.5	0–62.9	0	6.7	3.2–7.9	0
*R. gnavus*	4	0.2–27.0	0	4.4	0.1–26.9	0	10.5	6.0–12.4 ^2^	1.3
*C. hiranonis*	2.5	0.01–36.9	0	2.5	0–36.7	0	6.3	2.5–7.7	0
*Prevotella copri*	1.6	0–73.5	20.7	1.8	0–75.3	16.9	14	9.5–16.8 ^2^	9.0
*Faecalibacterium*	0.13	0–3.7	7.7	0.13	0–4.1	1.3	6.4	3.1–8.0	0
*Streptococcus*	0.07	0–61.8	28.2	0.07	0–61.4	6.5	7.8	1.1–8.7	1.3
*Lactobacillus*	0.03	0–88.5	29.5	0.02	0–88.1	10.5	N/A ^3^	N/A	N/A
*Bifidobacterium*	0.02	0–61.9	35.9	0.01	0–62.7	13.1	4	2.1–7.6	0
*Fusobacterium*	0.02	0–44.4	34.6	0.04	0–44.4	22.3	9.1	6.4–10.8	0
*Turicibacter*	0.01	0–2.3	33.3	0.02	0–2.5	10.5	6.8	4.3–9.0	0
*Escherichia coli*	0	0–4.7	67.9	0	0–5.0	52.6	4.7	0.9–7.7	3.8

^1^ UDL = under detection limit. Percentage of samples that have 0 counts in the sequencing data. Percentage of samples that have Cq-value of 40 in the qPCR assays. ^2^ Quantitative PCR was performed in 37 healthy dogs. ^3^ N/A = not applicable, *Lactobacillus* was not quantified by qPCR.

**Table 7:** Descriptive data (median and range) of the relative abundances of species (%) detected in the feces of all healthy dogs (*n* = 76) by shotgun metagenomic sequencing at rarefaction depth of 100,000.

Species	Median (%)	Range (%)
unclassified species in the family ***Lachnospiraceae***	5.1	0.04–29.7
*Ruminococcus gnavus*	4.4	0.13–26.9
*Clostridium hiranonis*	2.5	0.01–36.7
unclassified species in the order Bacteroidales	0.6	0.001–8.1
*Clostridium* sp. AT4	0.6	0.001–13.3
unclassified species in the order Clostridiales	0.5	0.03–2.2
unclassified species in the genus ***Bacteroides***	0.5	0.002–17.0
unclassified species in the phylum Firmicutes	0.3	0.02–1.1
unclassified species	0.3	0.01–1.0
*Coprococcus* sp. HPP0074	0.2	0.002–1.7
unclassified species in the genus ***Blautia***	0.2	0.02–11.8
*Blautia* sp. Marseille P3201T	0.2	0.002–1.3
*Clostridium glycyrrhizinilyticum*	0.1	0.001–0.6
*Blautia wexlerae*	0.05	0.004–6.096
*Blautia massiliensis*	0.04	0.001–0.251
*Blautia obeum*	0.03	0.002–0.848
*Fusicatenibacter saccharivorans*	0.01	0.001–0.251

## Data Availability

Metagenomic sequences are available under BioProject ID PRJNA975215.

## Supplementary Materials

The following supporting information can be downloaded at: https://www.mdpi.com/article/10.3390/ani13162597/s1, Figure S1. Beta diversity based on Bray–Curtis distances in different phenotypes; Figure S2. Observed features (alpha diversity) in different phenotypes; Table S1: Study population; Table S2: Quantitative PCR conditions of all targeted bacterial taxa in this study.

## References

[1] Bai H., Liu T., Wang S., Shen L., Wang Z. (2023). Variations in gut microbiome and metabolites of dogs with acute diarrhea in poodles and Labrador retrievers. Arch. Microbiol..

[2] Herstad K.M.V., Trosvik P., Haaland A.H., Haverkamp T.H.A., de Muinck E.J., Skancke E. (2021). Changes in the fecal microbiota in dogs with acute hemorrhagic diarrhea during an outbreak in Norway. J. Vet. Intern. Med..

[3] Minamoto Y., Otoni C.C., Steelman S.M., Büyükleblebici O., Steiner J.M., Jergens A.E., Suchodolski J.S. (2015). Alteration of the fecal microbiota and serum metabolite profiles in dogs with idiopathic inflammatory bowel disease. Gut Microbes.

[4] Xenoulis P.G., Palculict B., Allenspach K., Steiner J.M., Van House A.M., Suchodolski J.S. (2008). Molecular-phylogenetic characterization of microbial communities imbalances in the small intestine of dogs with inflammatory bowel disease. FEMS Microbiol. Ecol..

[5] Bharti R., Grimm D.G. (2021). Current challenges and best-practice protocols for microbiome analysis. Brief. Bioinform..

[6] Jovel J., Patterson J., Wang W., Hotte N., O’Keefe S., Mitchel T., Perry T., Kao D., Mason A.L., Madsen K.L. (2016). Characterization of the gut microbiome using 16S or shotgun metagenomics. Front. Microbiol..

[7] Atherly T., Rossi G., White R., Seo Y.J., Wang C., Ackermann M., Breuer M., Allenspach K., Mochel J.P., Jergens A.E. (2019). Glucocorticoid and dietary effects on mucosal microbiota in canine inflammatory bowel disease. PLoS ONE.

[8] D’Amore R., Ijaz U.Z., Schirmer M., Kenny J.G., Gregory R., Darby A.C., Shakya M., Podar M., Quince C., Hall N. (2016). A comprehensive benchmarking study of protocols and sequencing platforms for 16S rRNA community profiling. BMC Genom..

[9] Engelbrektson A., Kunin V., Wrighton K.C., Zvenigorodsky N., Chen F., Ochman H., Hugenholtz P. (2010). Experimental factors affecting PCR-based estimates of microbial species richness and evenness. ISME J..

[10] Tremblay J., Singh K., Fern A., Kirton E.S., He S., Woyke T., Lee J., Chen F., Dangl J.L., Tringe S.G. (2015). Primer and platform effects on 16S rRNA tag sequencing. Front. Microbiol..

[11] Quince C., Walker A.W., Simpson J.T., Loman N.J., Segata N. (2017). Shotgun metagenomics, from sampling to analysis. Nat. Biotechnol..

[12] Lewis S., Nash A., Li Q., Ahn T.-H. (2021). Comparison of 16S and whole genome dog microbiomes using machine learning. BioData Min..

[13] Tanprasertsuk J., Jha A.R., Shmalberg J., Jones R.B., Perry L.M., Maughan H., Honaker R.W. (2021). The microbiota of healthy dogs demonstrates individualized responses to synbiotic supplementation in a randomized controlled trial. Anim. Microbiome.

[14] Knight R., Vrbanac A., Taylor B.C., Aksenov A., Callewaert C., Debelius J., Gonzalez A., Kosciolek T., McCall L.I., McDonald D. (2018). Best practices for analysing microbiomes. Nat. Rev. Microbiol..

[15] Costea P.I., Zeller G., Sunagawa S., Pelletier E., Alberti A., Levenez F., Tramontano M., Driessen M., Hercog R., Jung F.-E. (2017). Towards standards for human fecal sample processing in metagenomic studies. Nat. Biotechnol..

[16] AlShawaqfeh M.K., Wajid B., Minamoto Y., Markel M., Lidbury J.A., Steiner J.M., Serpedin E., Suchodolski J.S. (2017). A dysbiosis index to assess microbial changes in fecal samples of dogs with chronic inflammatory enteropathy. FEMS Microbiol. Ecol..

[17] Felix A.P., Souza C.M.M., de Oliveira S.G. (2022). Biomarkers of gastrointestinal functionality in dogs: A systematic review and meta-analysis. Anim. Feed Sci. Technol..

[18] Werner M., Suchodolski J.S., Straubinger R.K., Wolf G., Steiner J.M., Lidbury J.A., Neuerer F., Hartmann K., Unterer S. (2020). Effect of amoxicillin-clavulanic acid on clinical scores, intestinal microbiome, and amoxicillin-resistant *Escherichia coli* in dogs with uncomplicated acute diarrhea. J. Vet. Intern. Med..

[19] Toresson L., Spillmann T., Pilla R., Ludvigsson U., Hellgren J., Olmedal G., Suchodolski J.S. (2023). Clinical effects of faecal microbiota transplantation as adjunctive therapy in dogs with chronic enteropathies—A retrospective case series of 41 Dogs. Vet. Sci..

[20] Pilla R., Gaschen F.P., Barr J.W., Olson E., Honneffer J., Guard B.C., Blake A.B., Villanueva D., Khattab M.R., AlShawaqfeh M.K. (2020). Effects of metronidazole on the fecal microbiome and metabolome in healthy dogs. J. Vet. Intern. Med..

[21] Belchik S.E., Oba P.M., Wyss R., Asare P.T., Vidal S., Miao Y., Adesokan Y., Suchodolski J.S., Swanson K.S. (2023). Effects of a milk oligosaccharide biosimilar on fecal characteristics, microbiota, and bile acid, calprotectin, and immunoglobulin concentrations of healthy adult dogs treated with metronidazole. J. Anim. Sci..

[22] Sung C.H., Marsilio S., Chow B., Zornow K.A., Slovak J.E., Pilla R., Lidbury J.A., Steiner J.M., Park S.Y., Hong M.P. (2022). Dysbiosis index to evaluate the fecal microbiota in healthy cats and cats with chronic enteropathies. J. Feline Med. Surg..

[23] Verbrugghe P., Van Aken O., Hallenius F., Nilsson A. (2021). Development of a real-time quantitative PCR method for detection and quantification of *Prevotella copri*. BMC Microbiol..

[24] Sokol H., Pigneur B., Watterlot L., Lakhdari O., Bermúdez-Humarán L.G., Gratadoux J.-J., Blugeon S., Bridonneau C., Furet J.-P., Corthier G. (2008). *Faecalibacterium prausnitzii* is an anti-inflammatory commensal bacterium identified by gut microbiota analysis of Crohn disease patients. Proc. Natl. Acad. Sci. USA.

[25] Brennan C.A., Clay S.L., Lavoie S.L., Bae S., Lang J.K., Fonseca-Pereira D., Rosinski K.G., Ou N., Glickman J.N., Garrett W.S. (2021). *Fusobacterium nucleatum* drives a pro-inflammatory intestinal microenvironment through metabolite receptor-dependent modulation of IL-17 expression. Gut Microbes.

[26] Pilla R., Suchodolski J.S. (2019). The role of the canine gut microbiome and metabolome in health and gastrointestinal disease. Front. Vet. Sci..

[27] Weingarden A.R., Dosa P.I., DeWinter E., Steer C.J., Shaughnessy M.K., Johnson J.R., Khoruts A., Sadowsky M.J. (2016). Changes in colonic bile acid composition following fecal microbiota transplantation are sufficient to control *Clostridium difficile* germination and growth. PLoS ONE.

[28] Blake A.B., Cigarroa A., Klein H.L., Khattab M.R., Keating T., Van De Coevering P., Lidbury J.A., Steiner J.M., Suchodolski J.S. (2020). Developmental stages in microbiota, bile acids, and clostridial species in healthy puppies. J. Vet. Intern. Med..

[29] Theriot C.M., Bowman A.A., Young V.B. (2016). Antibiotic-induced alterations of the gut microbiota alter secondary bile acid production and allow for *Clostridium difficile* spore germination and outgrowth in the large intestine. mSphere.

[30] Thanissery R., Winston J.A., Theriot C.M. (2017). Inhibition of spore germination, growth, and toxin activity of clinically relevant *C. difficile* strains by gut microbiota derived secondary bile acids. Anaerobe.

[31] Wang S., Martins R., Sullivan M.C., Friedman E.S., Misic A.M., El-Fahmawi A., De Martinis E.C.P., O’Brien K., Chen Y., Bradley C. (2019). Diet-induced remission in chronic enteropathy is associated with altered microbial community structure and synthesis of secondary bile acids. Microbiome.

[32] Guard B.C., Honneffer J.B., Jergens A.E., Jonika M.M., Toresson L., Lawrence Y.A., Webb C.B., Hill S., Lidbury J.A., Steiner J.M. (2019). Longitudinal assessment of microbial dysbiosis, fecal unconjugated bile acid concentrations, and disease activity in dogs with steroid-responsive chronic inflammatory enteropathy. J. Vet. Intern. Med..

[33] Blake A.B., Guard B.C., Honneffer J.B., Lidbury J.A., Steiner J.M., Suchodolski J.S. (2019). Altered microbiota, fecal lactate, and fecal bile acids in dogs with gastrointestinal disease. PLoS ONE.

[34] Manchester A.C., Webb C.B., Blake A.B., Sarwar F., Lidbury J.A., Steiner J.M., Suchodolski J.S. (2019). Long-term impact of tylosin on fecal microbiota and fecal bile acids of healthy dogs. J. Vet. Intern. Med..

[35] Duboc H., Rajca S., Rainteau D., Benarous D., Maubert M.A., Quervain E., Thomas G., Barbu V., Humbert L., Despras G. (2013). Connecting dysbiosis, bile-acid dysmetabolism and gut inflammation in inflammatory bowel diseases. Gut.

[36] Li Q., Larouche-Lebel É., Loughran K.A., Huh T.P., Suchodolski J.S., Oyama M.A. (2021). Gut dysbiosis and its associations with gut microbiota-derived metabolites in dogs with myxomatous mitral valve disease. Msystems.

[37] Minamoto Y., Minamoto T., Isaiah A., Sattasathuchana P., Buono A., Rangachari V.R., McNeely I.H., Lidbury J., Steiner J.M., Suchodolski J.S. (2019). Fecal short-chain fatty acid concentrations and dysbiosis in dogs with chronic enteropathy. J. Vet. Intern. Med..

[38] Giaretta P.R., Suchodolski J.S., Jergens A.E., Steiner J.M., Lidbury J.A., Cook A.K., Hanifeh M., Spillmann T., Kilpinen S., Syrja P. (2020). Bacterial Biogeography of the Colon in Dogs With Chronic Inflammatory Enteropathy. Vet. Pathol..

[39] Pilla R., Guard B.C., Blake A.B., Ackermann M., Webb C., Hill S., Lidbury J.A., Steiner J.M., Jergens A.E., Suchodolski J.S. (2021). Long-term recovery of the fecal microbiome and metabolome of dogs with steroid-responsive enteropathy. Animals.

[40] Weiss S., Xu Z.Z., Peddada S., Amir A., Bittinger K., Gonzalez A., Lozupone C., Zaneveld J.R., Vázquez-Baeza Y., Birmingham A. (2017). Normalization and microbial differential abundance strategies depend upon data characteristics. Microbiome.

[41] Vandeputte D., Kathagen G., D’Hoe K., Vieira-Silva S., Valles-Colomer M., Sabino J., Wang J., Tito R.Y., De Commer L., Darzi Y. (2017). Quantitative microbiome profiling links gut community variation to microbial load. Nature.

[42] Willis A.D. (2019). Rarefaction, alpha diversity, and statistics. Front. Microbiol..

[43] McMurdie P.J., Holmes S. (2014). Waste not, want not: Why rarefying microbiome data is inadmissible. PLoS Comput. Biol..

[44] Hillmann B., Al-Ghalith G.A., Shields-Cutler R.R., Zhu Q., Gohl D.M., Beckman K.B., Knight R., Knights D. (2018). Evaluating the information content of shallow shotgun metagenomics. Msystems.

