# Supplementary material for: Correlation between Targeted qPCR Assays and Untargeted DNA Shotgun Metagenomic Sequencing for Assessing the Fecal Microbiota in Dogs
Source: Animals (Basel). 2023 Aug 11;13(16):2597. doi: 10.3390/ani13162597 (PMC10451198; doi:10.3390/ani13162597)
Supplement: Supplementary file 1 [file animals-13-02597-s001.zip › animals-2521648-supplementary.pdf]

Supplementary Figure S1. Beta diversity based on Bray-Curtis distances in different phenotypes.

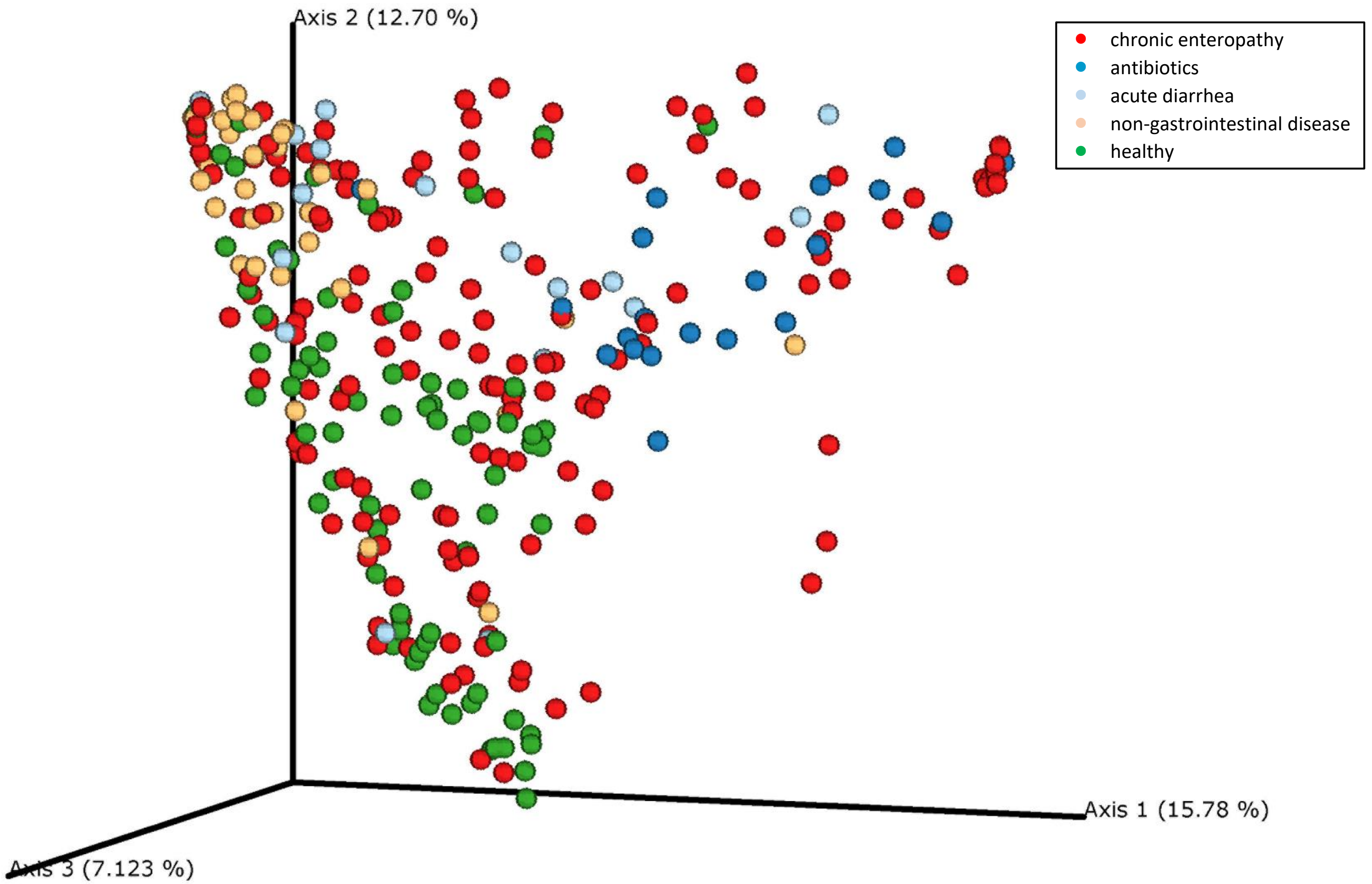

Compared to healthy control	R value	P value
chronic enteropathy	0.015	0.23
acute diarrhea	0.385	0.001
on antibiotics	0.721	0.001
non-gastrointestinal disease	0.074	0.05

Supplementary Figure S2. Observed features (alpha diversity) in different phenotypes. Kruskal-Wallis test followed by Dunn's multiple comparison tests was applied. No differences were found between healthy and dogs with all other disease phenotypes. Dogs receiving antibiotics had significantly lower observed features compared to clinically healthy dogs.

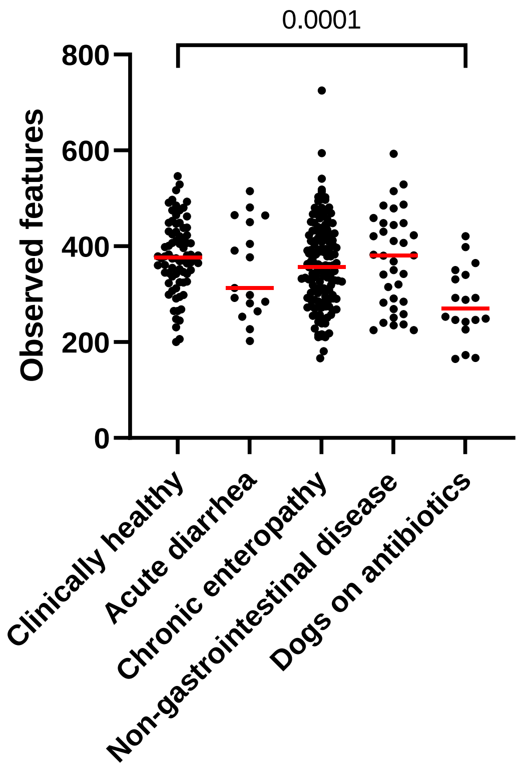

Supplementary Table S1. Study population.

Group	Total	Inclusion criteria	Institution/Hospital
Clinically healthy	78	• clinically healthy for at least one year without any gastrointestinal signs, no history of systemic disease<br>• did not receive any antibiotics, antacids, anti-inflammatory medications, or corticosteroids within the past 6 months	• Evidensia Specialist Animal Hospital in Helsingborg, Sweden<br>• Iowa State University<br>• Ludwig-Maximilians University, Munich, Germany<br>• Texas A&M University<br>• Vetsuisse Faculty, Zurich, Switzerland<br>• University of Illinois at Urbana-Champaign<br>• University of Veterinary Medicine, Hannover, Germany
Chronic enteropathy	146	• a history of persistent or intermittent clinical signs of GI disease for a minimum of 3 weeks<br>• any recorded antibiotic exposure was excluded from this group<br>• exclusion of extra-intestinal disease with ultrasonography, clinical chemistry, including basal cortisol, hematology, parasitology, and histopathologic evidence of GI mucosal inflammation	• Evidensia Specialist Animal Hospital in Helsingborg, Sweden<br>• Iowa State University<br>• Oregon State University<br>• Texas A&M University<br>• Vetsuisse Faculty, Zurich, Switzerland
Acute diarrhea	17	• a duration of acute non-hemorrhagic diarrhea < 3 days<br>• fecal samples were collected upon admission, prior to treatments	• Ludwig-Maximilians University, Munich, Germany
Non-gastrointestinal disease	35	• owners of drug-naïve dogs with idiopathic epilepsy<br>• histopathologically confirmed non-alimentary mast cell tumor, melanoma, or osteosarcoma	• University of Veterinary Medicine, Hannover, Germany<br>• Small Animal Clinic of Veterinary Faculty Ljubljana, Slovenia
On antibiotics	20	• healthy dogs received metronidazole in experimental studies	• Louisiana State University<br>• University of Illinois at Urbana-Champaign<br>• University of Veterinary Medicine, Hannover, Germany

Supplementary Table S2. Conditions of qPCR assays using in this study.

Target	Primer*	Amplicon length(bp)	Annealing temperature (°C)	Melting temperature (°C)	Efficiency (%)	R^2	Reference
Bacteroides	TCGGCTAACTCCGTGCCAGC<br>ACACCACGAATTCCGCCCACC	184	52	84.0	92.4	0.995	Sung [1]
Bifidobacterium	TCGCGTCYGGTGTGAAAG<br>CCACATCCAGCRTCCAC	243	60	86.5	99.2	0.998	Sung [1]
Blautia	TCTGATGTGAAAGGCTGGGGCTTA<br>GGCTTAGCCACCCGACACCTA	250	56	84.0	91.9	0.996	AlShawaqfeh [2]
Clostridium hiranonis	AGTAAGCTCCTGATACTGTCT<br>AGGGAAAGAGGAGATTAGTCC	401	59	85.5	96.2	0.995	AlShawaqfeh [2]
Collinsella	GGTAATACCCGATGACCCCG<br>TCTCATCCCTGCTGAAAGCG	288	60.3	90.5	97.0	0.999	Designed in house
Escherichia coli	GTTAATACCTTTGCTCATTGA<br>ACCAGGGTATCTAATCCTGTT	340	55	86.5	99.5	0.997	AlShawaqfeh [2]
Faecalibacterium	GAAGGCGGCCTACTGGGCAC<br>GTGCAGGCGAGTTGCAGCCT	597	60	85.5	92.9	0.994	AlShawaqfeh [2]
Fusobacteria	KGGGCTCAACMCMGTATTGCGT<br>TCGCGTTAGCTTGGGCGCTG	253	50.5	84.0	100.2	0.991	AlShawaqfeh [2]
Prevotella corpi	ACCACTTGGGGATAACCTTG<br>TACATGCAAAAAGCCTCACGAGGC	347	58.4	85.0	100.5	0.994	Verbrugghe [3]
Ruminococcus gnavus	CATCTGAATCGCCGCCTTTG<br>CCTGCGTTACGATCAAAGCG	350	60	86.5	95.8	0.998	Designed in house
Streptococcus	TTATTTGAAAGGGGCAATTGCT<br>GTGAACTTTCCACTCTCACAC	279	54	87.0	100.3	0.992	AlShawaqfeh [2]

Turicibacter	CAGACGGGGACAACGATTGGA<br>TACGCATCGTCGCCTTGGTA	140	57	83.0	90.0	0.998	AlShawaqfeh [2]
Universal bacteria	CCTACGGGAGGCAGCAGT<br>ATTACCGCGGCTGCTGG	177	59	83.0	98	0.998	AlShawaqfeh [2]

*Primer: forward primer on the top, reverse primer at the bottom

1. Sung, C.H.; Marsilio, S.; Chow, B.; Zornow, K.A.; Slovak, J.E.; Pilla, R.; Lidbury, J.A.; Steiner, J.M.; Park, S.Y.; Hong, M.P.; et al. Dysbiosis index to evaluate the fecal microbiota in healthy cats and cats with chronic enteropathies. *J Feline Med Surg* **2022**, *24*, e1-e12.
2. AlShawaqfeh, M.K.; Wajid, B.; Minamoto, Y.; Markel, M.; Lidbury, J.A.; Steiner, J.M.; Serpedin, E.; Suchodolski, J.S. A dysbiosis index to assess microbial changes in fecal samples of dogs with chronic inflammatory enteropathy. *FEMS Microbiol Ecol* **2017**, *93*.
3. Verbrugghe, P.; Van Aken, O.; Hallenius, F.; Nilsson, A. Development of a real-time quantitative PCR method for detection and quantification of Prevotella copri. *BMC Microbiol* **2021**, *21*, 23.
